# Maryland and District of Columbia Dental Association

**Published:** 1878-12

**Authors:** 


AMERICAN JOURNAL

OF

DENTAL SCIENCE.

Vol. XII. THIRD SERIES—DECEMBER, 1878. No. 8.

### ARTICLE I.

### *Maryland and District of Columbia Dental Association.*

(Continued.)

[Reported for this Journal.]

At the conclusion of the reading of the report of the Section on Anæsthesia, the subject was discussed by Dr. Turnbull, of Philadelphia, who said that he was present more as a listener than as a debater, and he felt after the reading of the exhaustive essay that those present knew probably more about the subject than himself. Still he would say a word. He viewed with amazement the statistics of Prof. Chisolm. He did not question his authority. His thousands of cases and no deaths had the air of authority, and his summary of only twelve deaths from chloroform he had no doubt, was honestly avered. But it so happened that in our own land alone there were a great many more deaths than this. It is only recently that cases have been published. So far there was no official record of cases of deaths in the Army or Navy of the U. S. The truth is we are loth, as surgeons, to tell the deaths from Anæsthetics—we are slow to say that people die of chloroform. 210 deaths were reported in "Eely's *Medical News and Library*" alone, and the current reports show that in 1869, there were 21 deaths from chloroform, in 1870, 9 deaths; in 1871, 10 deaths; in 1872, 15 deaths; in 1873, 14 deaths; in 1874, 9 deaths; in 1875, 5 deaths; in 1876, 4 or 5 deaths; in 1877, 6 deaths; in 1878, 4 deaths; and yet in the face of these figures, surgeons say chloroform is safe!

Dr. Hunter McGuire had boasted that he had never had an accident, but only very lately he saw it reported that even he had had a fatal casualty.

He discussed the action of Nitrate of Amyl, and thought its action on the heart was secondary. The action of chloroform on an eel in water, showed that it would produce death. If it were given as an anæsthetic, it should be very largely diluted; at least 96 parts of air. There was an accumulation of the anæsthetic in the system, which was dangerous. He considered it specially contra-indicated in kidney disease. Where chloroform and ether were mixed, it should be in the proportion of one part of chloroform to three of ether, by *weight.* Of course it would be impossible to give pure ether vapor, though the mouth should be closely covered. Some air will get in. As to the action of these agents on the blood, it was a matter of doubt if it were understood. Some modification of protoplasm was no doubt made, and their effects on muscular tissue peculiar. Chloroform coagulates albumen, and coagulated albumen cannot be restored. This is a fact which should caution us in its use. In extremely hot countries it was necessary to use it on account of the low boiling point of ether. With regard to the theories of Dr. Waterman, as to the changes seen in blood in the spectroscopic analysis, he thought they were not well founded, and no change of the grave sort had been seen under the microscope. He thought the chemists who made the Nitrate of Ammonia were not reliable always, and he was informed by a person who had great reputation for the purity and success of the gas he used, that he had great trouble in obtaining a pure article of the salt from which the gas is generated. This informant stated that he based his success on generating a pure article of gas, and of always having it fresh, and never administering it until it had stood at least seven hours, and never using it when more than a few weeks old. The only catastrophe he had heard of was the swallowing of a cork put between the patient's teeth, which got into the trachea and produced death. The action of Nitrous Oxide seems first a shock, and then flushes the face, and then produces muscular contraction. The few cases of death set down to its use were so slight as to be almost nothing.

As to ether, the statistics in this country and in England were unequal as to the ratio of deaths. In England in 1876–7 there were 18 deaths from its use, while in America during the same period there were only 3 or 4 with us. We were careful to use a pure article, while in England the drug was often adulterated or impure. He was understood to say that ether was alcohol minus water, the effect of the Sulphuric Acid used in its manufacture being to remove the water.

Dr. Atkinson asked, but did not get a satisfactory reply as to this statement respecting ether. Water, he said was used to purify the ether and to wash it—and if it had any affinity for that fluid, it would surely show it.

Adjourned.

AFTERNOON.

Dr. Turnbull further discussed the question of anæsthesia, and of nitrous oxide gas, expressing himself in favor of the condensed gas as manufactured more uniformly and as not deteriorating with age. Prof. Barker had showed the injurious effects of this gas years ago; and it was also demonstrated that the old gas of the gasometers was inoperative. In Philadelphia, most of the gas is given by one man, and most of those who need extracting done by its use, patron ize one dentist.

Dr. Ten Eyck, of Washington, D. C., said that when in Philadelphia, he had visited the office of the dentist in question, (Dr. Thomas,) and he had informed him that he did not consider gas fit to inhale after two days. He had given it to 185,000 persons, never refusing except in two cases, both of whom died a few days after he had declined to serve them. The impurities in the gas as ordinarily made, he inferred were produced by the impurity of the Nitrate of Ammonia.

Prof. Hodgkin said, that when gas was first introduced he had used it somewhat freely, with no observable bad effects, but that since the paper of Dr. Waterman had been published he had felt uneasy for fear he had unwittingly hurried some of his patients on their road over the river. He did not give gas now, mainly because there seemed to be but little demand for it, and partly on account of the trouble of it. As to the fact alleged that the gas could not be kept more than 48 hours, he did not believe it, as all who know anything about it, know that it is one of the most stable of the gaseous compounds; and he was sure that the only deterioration it could suffer, would be the dilution with air, passing through the water of the gasometer. He would say while he was up, that interested in the subject as a journalist, he had sent out a great many circulars to physicians, and had an appeal widely published, asking for information as to these after effects of nitrous oxide gas, and requesting any physician who might have noticed any such injurious effects to communicate them. But one response—that one eulogistic of the gas—had been received, which would hardly have been the case if the effects had been so deleterious as alleged by Dr. Waterman in his paper.

Dr. J. J. Caldwell, of Baltimore, said that he was an M. D., and knew but little about the subject of gas. He thought, however, that the dentists were the only men to note the effects, if any injury was done. The physician would probably not have his attention excited in that direction of enquiry. He thought that Waterman's enthusiasm had carried him rather beyond the bounds of reason. He did not believe in this "death lime" which his friend Waterman saw in the spectroscope. Still, no anæsthesia is safe. Any such condition is a step toward death, which we should hesitate to take. He did not believe that change in protoplasm could be detected after death; but we must see how dangerous is the condition of one with weak heart and lungs, and with the spinal cord overwhelmed with anæsthesia. The sympathetic is left to carry on life of itself, and if it is feeble, what is to keep up the life? Who knows anything of the pathology of the sympathetic? But if, as the report of the section says, the two legs of the tripod of Bichat are taken away, what has the vital powers left to lean on? We are indeed gravely simulating death, which is too apt to prove a ghastly reality on our hands.

Dr. R. F. Hunt, of Washington, D. C., asked for information as to the specific effect of the mixture of two kinds of anæsthetics, as ether and chloroform,—as for example when ether is given for awhile and then laid aside and the other given? He cited the analogy between the effect of single acids on metal and their combination, and the well-known effects of "mixed drinks" in producing a more formidable intoxication than could be brought about by single fluids of the sort. He thought that carefully conducted experiments ought to be made to determine these points.

Dr. Turnbull replied that so far as was known, on mixing two parts of chloroform with one part of ether, a chemical change was produced, there was a condensation of the liquids and a new chemical compound. In all administrations of this drug it was considered important that whoever gave the ether should do nothing else. The vapor was heavy and fell to the floor, and being inflammable should never be used where there was a light, or near a fire. Hydro-bromic ether was an excellent anæsthetic, but was costly. It was worth $4 per pound.

Dr. Caldwell. Babies take chloroform as they do milk; it seems not to injure them at all. He spoke of the great importance of electricity in case of trouble from an anæsthetic. He thought that we have in a battery a swift and direct remedy. When at the lowest ebb of life, the current may be applied, and life saved. He cited the case of a boy who had all the help in anæsthesia he could have—Squibbs, Sims, Sayer, were present, but fatal symptoms came on and he succumbed. Had a battery been on hand, he might have been saved.

Dr. E. Cutter, of Boston, exhibited an exceedingly simple and ingenious apparatus for the resuscitation of apparently drowned people. It consisted simply of two open cans fitting very loosely, one into the other, the inner one with a projecting pipe for the attachment of an india-rubber hose. The whole affair not unlike an ordinary gasometer for nitrous oxide gas on a small scale. The outer tank could be dispensed with, if the asphyxiated person were at the water side, and the small or inner tank plunged into the river, lake or body of water from which the body had been rescued.

Dr. Turnbull, in reply to a question as to what he thought of the method proposed by Dr. M. H. Webb, of rapid breathing as an anæsthetic, said it was one of a train of nervous phenomena akin to the "metallic tractors" of former days.

Dr. Cutter gave his method for inhaling ether. It was to spread a towel over the face and drop the drug upon this. Very little was sufficient to produce the effect. Death is caused in such cases from the retention of carbonic acid in the lungs. The simple apparatus he presented would remove this and supply pure air.

Dr. Coy, of Baltimore, thought the condensed gas purer than that ordinarily made in the dentists' office. He thought much of the quiet conduct to be desired on the part of the patient was to be brought about by being quiet and cool ourselves. Any excitement or emotion on our part would be sure to be communicated to the patient. He always gave along with the chloroform, plenty of air at first, and insisted on the horizontal position. Occasionally it might be seen from the behavior of the patient that the chloroform was not acting well, in which case he substituted ether. He did not consider this following of drugs, one by the other, was equivalent to mixing them. And he sometimes found it best to reverse the procedure, and give chloroform after the ether. He thought that anæsthetics did not lower the tone of the system as much as did the surgical shock.

Dr. Atkinson placed himself on the record by declaring chloroform to be satan's prime minister, especially among progeny; and nitrate oxide gas ranked next. He criticised the theory of Dr. Cutter, as to asphyxia from drowning, and claimed that the lungs were not empty, as claimed, but that the air was then fast locked, and that under no circumstances could more than four per cent. of the oxygen contained in the respired air be used up by the lungs, the rest being rendered unfit for use. He characterized this discussion as that of men full of the confidence of ignorance,—a set of asses in council. "Anæsthesia is privity of oxygen."

Dr. Cutter took exception to this, and contended that if this were true then C O2 would be a true anæsthetic.

Dr. Turnbull argued that C O2 would produce local anæsthesia, and that carcinomatous masses had been removed whilst the patient was under its influence.

Dr. Caldwell. We do have anæsthesia from carbonic acid gas. This was known long ago, and it has been for years in France a popular method of suicide. We need not know, as seems to be insisted upon by some, what anæsthesia is, before we have anæsthetics. But it cannot be that it is privity of oxygen, for we have this condition with abundant access of air, and with respiration unimpeded.

After further discussion the subject was passed,

On motion of Dr. H. H. Keech, of Baltimore, Thomas

Morrow, of that city, was appointed the State student to the Maryland Dental College.

Adjourned.

THIRD DAY.—Morning.

After some routine business, on motion of Dr. Hunt, of Washington, D. C., so much of the annual address of the President of the Association, as related to the subject of the Goodyear Dental Vulcanite Company, and their suits against the dentists, was referred to a special committee; Drs. Winder, Smithe and H. H. Keech, being appointed as the committee.

The report of the Section on Pathology and Therapeutics was read. It embraced a paper by Dr. Caldwell, on alcoholism and another on syphilis.

A recess was then taken and the canvassing of subscriptions to aid Dr. Hunt in the prosecution of the rubber suits was carried on, Dr. Winder and others setting forth the claims of Dr. Hunt to the favorable consideration of the association, he having given all his time and means to the furtherance of these matters, and had labored as few men would have done. Dr. Winder gave a summary of the condition of the suit, and a resumè of the evidence so far collected, which it was now designed to print.

On motion of Dr. Waters, it was resolved that all papers read before the Association be considered the property of the Association; and that any paper presented and read here, and afterwards published by the writer previously to its appearance in the published proceedings of the society, shall not be printed in such proceedings.

A discussion followed, in which it was elicited that the Association did not object to having an abstract made of the proceedings, but that they rather desired this.

The subject of Pathology and Therapeutics was passed, and Artificial Dentistry taken up.

Dr. W. W. Evans read the report of the Section. The ground taken in the paper was that the idea that operative dentistry alone was necessary to success was a fatal error, that students should be urged to study dental art. In this the Colleges are at fault, not developing this branch of the profession as it should be, and as it deserves.

The report was discussed by Dr. Hunt, who thought that Artificial Dentistry ought to be viewed in an æsthetic light, and placed in the same plane with operative dentistry. It was sadly neglected of late years and had retrograded. It was the duty of the profession to stimulate the manufacturers of porcelain teeth to improvement in naturalness; in their productions with the millions of teeth on hand, it is really difficult to get good cases. The teeth made are so little like nature. No part of our art had sunk so low, and was so degraded as this.

Dr. Noble thought that a higher degree of talent was required for this branch of dentistry than was generally supposed. Its difficulty and the taste and knowledge required was not generally recognized. Many of those wearing artificial teeth are simply disfigured by them.

Dr. R. B. Donaldson, of Washington, said that one thing was absolutely necessary, before the advancement spoken of as so desirable could be made—the education of the public in matters of this sort. The mass of those requiring artificial teeth had not the slightest appreciation of the merits of the subject, and fell into the hands of any who chose to impose on them. In the main this trouble rests with the public. People will not pay for a proper denture. Qualified dentists, who have qualified themselves by long years of careful study and close attention cannot and will not enter into competition with the cheap men. For himself he had carved and baked his own teeth for the first fifteen years of his practice, and his income from this branch alone had ranged from $6,000 to $7,000 a year. But during the past three years it had not amounted to $600 a year. People will not pay his charges for artistic work, and go elsewhere; and so the work of supplying artificial teeth to those who require them, falls into incompetent hands.

Dr. Coy said that the old operators in order to color teeth properly and understand shades, studied this subject from human models—studied the effects of light upon tooth structure in the mouth; the effects of gas light, &c. Often a tooth which appeared all right by daylight was seen to be out of place in shade at night. We should study the shade accompanying temperaments, the associated color of hair and complexion. He could seldom get selections made for him; and the fact that when he did go to the dental depot to select teeth, he found there boys and even colored servants selecting teeth for the dentist, showed how little appreciation some men had of the subject under discussion. Some of this fault—the degraded condition of this branch of dentistry—is ours, in taking work at too low figures.

Subject passed. Adjourned.

AFTERNOON.

The first subject in order was the election of officers for the ensuing year which resulted as follows:

*President.*—J. Curtiss Smithe, D. C.

*Vice President.*—M. W. Foster, Baltimore.

*Recording Secretary.*—H. M. Schooley, D. C.

*Corresponding Secretary.*—R. B. Winder, Baltimore.

*Reporting Secretary.*—T. S. Waters, Baltimore.

*Treasurer.*—H. B. Noble, D. C.

*Executive Committee.*—Drs. R. B. Winder, Chairman; and B. F. Coy, Baltimore, H. C. Thompson, D. C.

*Publication Committee.*—H. M Schooley, Chairman; and J. B. Ten Eyck, D. C.

Votes of thanks were passed to Rev. Dr. Elliot, and Drs. Turnbull and Cutter, for their presence and aid.

The report of the Section on Dental Education and Literature was read by Dr. Noble. It referred to the accidental origin of the oldest Dental College in the World, resulting from the refusal of the medical fraternity to offer opportunities for study of this branch of surgery and medicine, also to one of the youngest of the schools, the Maryland Dental College, which seeks thoroughness in preparation on the part of her graduates, carefully guarding against the graduation of any who are not fully qualified. The paper referred to a hope for the future union of these two institutions—the Baltimore College of Dental Surgery and the Maryland Dental College. The question of the final examination of students was discussed, and some suggestions made as to improvement in the methods of examination, and also in the appointment of professors. One method had been suggested, viz: that the State Societies elect men to fill vacancies, and another plan that the alumni nominate persons to fill such chairs. The opinion was expressed that the colleges could not do this work well unless free from their present poverty, and endowments were absolutely necessary; teaching in most of the schools bring a labor of love. The proposition that dental schools should be broken up in favor of medical schools was wrong, and should not be listened to. The universities had established dental schools, and the improvement of the opportunity for an advanced standard was anxiously looked for.

The subject was briefly discussed by Drs. Hunt, Donaldson, and others, and the regret expressed that it had not been brought up earlier in the session,—and then passed.

The installation of officers followed, and the Association adjourned to meet in Baltimore in 1879.

